# Α *de novo* 3.8-Mb inversion affecting the *EDA* and *XIST* genes in a heterozygous female calf with generalized hypohidrotic ectodermal dysplasia

**DOI:** 10.1186/s12864-019-6087-1

**Published:** 2019-09-18

**Authors:** Clémentine Escouflaire, Emmanuelle Rebours, Mathieu Charles, Sébastien Orellana, Margarita Cano, Julie Rivière, Cécile Grohs, Hélène Hayes, Aurélien Capitan

**Affiliations:** 1 INRA, GABI, AgroParisTech, Université Paris-Saclay, 78350 Jouy-en-Josas, France; 2 Allice, 75595 Paris, France; 3 INRA, SIGENAE, GABI, 78350 Jouy-en-Josas, France; 4 INRA, Micalis Institute, AgroParisTech, Université Paris-Saclay, 78350 Jouy-en-Josas, France

**Keywords:** Hypohidrotic ectodermal dysplasia, EDA, XIST, Whole-genome sequencing, Holstein, Cattle

## Abstract

**Background:**

In mammals, hypohidrotic ectodermal dysplasia (HED) is a genetic disorder that is characterized by sparse hair, tooth abnormalities, and defects in cutaneous glands. Only four genes, *EDA*, *EDAR*, *EDARADD* and *WNT10A* account for more than 90% of HED cases, and *EDA*, on chromosome X, is involved in 50% of the cases. In this study, we explored an isolated case of a female Holstein calf with symptoms similar to HED.

**Results:**

Clinical examination confirmed the diagnosis. The affected female showed homogeneous hypotrichosis and oligodontia as previously observed in bovine *EDAR* homozygous and *EDA* hemizygous mutants. Under light microscopy, the hair follicles were thinner and located higher in the dermis of the frontal skin in the affected animal than in the control. Moreover, the affected animal showed a five-fold increase in the number of hair follicles and a four-fold decrease in the diameter of the pilary canals. Pedigree analysis revealed that the coefficient of inbreeding of the affected calf (4.58%) was not higher than the average population inbreeding coefficient (4.59%). This animal had ten ancestors in its paternal and maternal lineages. By estimating the number of affected cases that would be expected if any of these common ancestors carried a recessive mutation, we concluded that, if they existed, other cases of HED should have been reported in France, which is not the case. Therefore, we assumed that the causal mutation was dominant and de novo. By analyzing whole-genome sequencing data, we identified a large chromosomal inversion with breakpoints located in the first introns of the *EDA* and *XIST* genes. Genotyping by PCR-electrophoresis the case and its parents allowed us to demonstrate the de novo origin of this inversion. Finally, using various sources of information we present a body of evidence that supports the hypothesis that this mutation is responsible for a skewed inactivation of X, and that only the normal X can be inactivated.

**Conclusions:**

In this article, we report a unique case of X-linked HED affected Holstein female calf with an assumed full inactivation of the normal X-chromosome, thus leading to a severe phenotype similar to that of hemizygous males.

## Background

In Mammals, hypohidrotic ectodermal dysplasia (HED) is a well-described genetic disorder that is characterized by sparse hair (hypotrichosis), abnormal or missing teeth (oligodontia), and reduced ability to sweat (hypohidrosis) [1]. Typical clinical features also include defective development of other exocrine glands, which causes dryness of the skin and mucosa, and occasionally absence of teats and dystrophic nails [2]. This condition is known in humans and represents the most frequent form of human ectodermal dysplasia, but has also been reported in a number of species such as rat [3], dog [4] and cattle [5]. Without adequate medical care, the life span of affected individuals is markedly reduced because of sensitivity to temperature fluctuations, respiratory problems and feeding difficulties [6, 7].

Studies in humans have demonstrated that only four genes (*EDA, EDAR*, *EDARADD*, and *WNT10A*) account for more than 90% of HED cases, and that *EDA* is involved in more than 50% of cases [8]. *WNT10A* encodes the wingless-type mouse mammary tumor virus (MMTV) integration site family member 10A (WNT10A), which is a small signaling protein belonging to the Wnt family. WNT10A is a key modulator of cell–cell interactions in many tissues, and also contributes to the embryonic development of skin, hair, nails and teeth [9–11]. *EDA*, *EDAR* and *EDARADD*, each encode one of the three proteins of the ectodysplasin pathway, namely ectodysplasin-A (EDA), which is a member of the tumor necrosis factor (TNF) superfamily, its receptor (EDAR), and a specific adaptor protein, the EDAR associated death domain (EDARADD) [12]. The ectodysplasin pathway functions downstream of the Wnt/β-catenin signaling and plays an essential role in the morphogenesis of ectodermal organs [13].

Mutations in *EDAR*, *EDARADD* and *WNT10A* are most frequently autosomal recessive, whereas mutations in EDA, which is located on the X chromosome, are inherited in a X-linked recessive pattern and cause the X-linked form of HED (XLHED) [14]. In contrast to males, females that are heterozygous for *EDA* mutations typically show less severe symptoms with a reduced degree of hypodontia, mosaic patterns of hypotrichosis, and defective sweat glands along Blaschko’s lines because of random X inactivation [15–17].

In 2014, a female calf that presented typical symptoms of HED was reported to the French National Observatory for Bovine Genetic Defects (ONAB, https://www.onab.fr/). Its parents were both phenotypically normal. ONAB has no other record of such cases in the French Holstein population during the period 2002–2018. This isolated case offered a rare opportunity to identify new genes and/or a new mode of inheritance involved in HED.

In this study, we report the identification of a 3.8-Mb inversion on chromosome X of a heterozygous female calf that causes a dominant and generalized form of HED via skewed X-inactivation and truncation of the EDA protein.

## Results

### Clinical examination

**Fig. 1:**
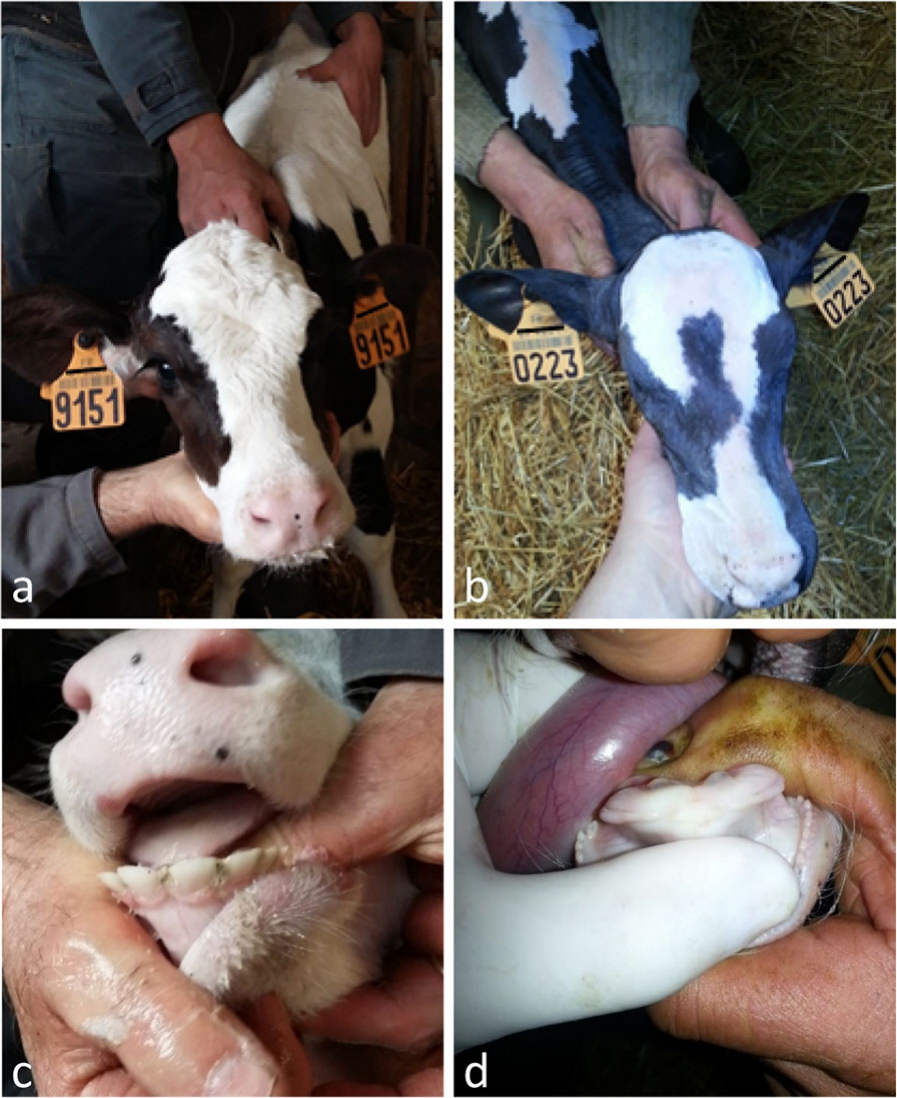
Pictures of a control animal (1 month old, **a-c**) and the affected animal (2 weeks, **b-d**). Please note the difference in hairiness between the two animals (**a-b**) and the absence of incisors in the affected calf (**d**)

**Fig. 2:**
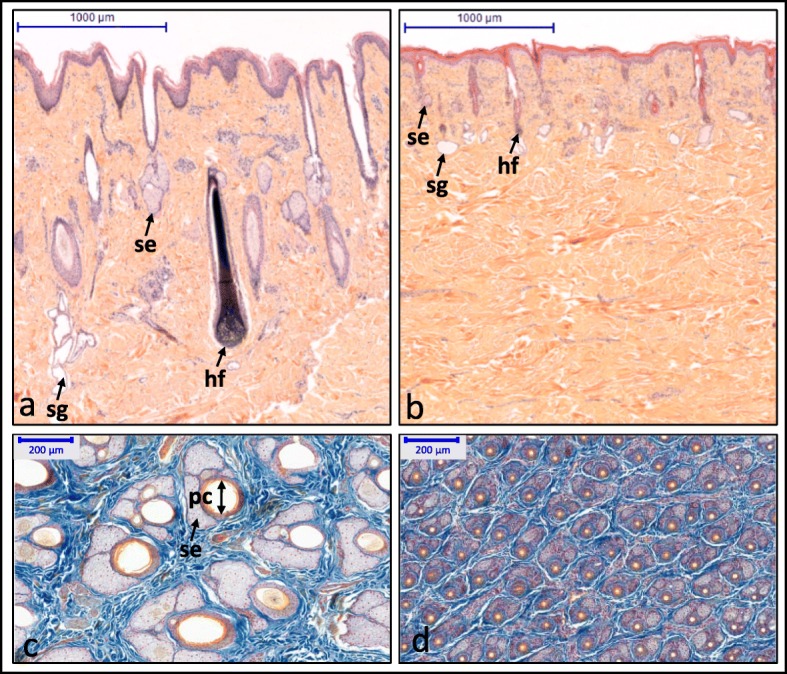
Sections of frontal skin samples from the affected calf and a control. Transversal sections of the skin of control (**a**) and HED animals (**b**) stained with hematoxylin-eosin-safran. Note the reduced size of sweat glands (sw), sebaceous glands (se), and hair follicles (hf) in the affected (**b**) versus control (**a**) animals. Longitudinal sections of the skin of control (**c**) and HED animals (**d**) stained with a Roan solution (Orange G, aniline blue staining and Kernechtrot). Note the increased density but reduced size of sebaceous glands (se), and pilary canals (pc) in the affected (**d**) versus control (**c**) animals

A Holstein female calf, conceived through artificial insemination and born after a normal gestation period of 275 days, was reported to the ONAB for abnormal hair coat. Hairs were short and thin over the whole body and tended to be slightly longer on the fetlocks and the tail. Whiskers were normal and horn buds appeared normal for its age (Fig. 1 and Additional file 5: Figure S1). Dental examination revealed a complete absence of teeth with the exception of one malformed premolar on each side of the upper jaw (Fig. 1 and Additional file 5: Figure S1 and Additional file 6: Figure S2). This animal died at 2 weeks of age of pulmonary infection. Unfortunately, we did not have the possibility to investigate its internal organs but obtained formalin-fixed skin samples from the forehead and horn bud areas for post-mortem histological analyses. Observation under light microscopy showed that, in the affected calf, the hair follicles were thinner and located higher in the dermis than in the control animal (Fig. 2a-b). Sebaceous and sweat glands were hypoplastic. Analysis of longitudinal skin sections demonstrated that there were five times more hair follicles in the frontal skin of the affected calf than the control (mean = 47.7 vs 9.3 hairs in 1-mm^2^ quadrats) and that the diameter of their pilary canals was four times smaller (mean = 21.6 vs 87.2 μm, Fig. 2c-d, Table 1). Histological analyses also revealed that the affected calf had normal horn buds (Additional file 7: Figure S3).

**Table 1:** Measurement of hair count, diameter of the pilary canal and area covered by pilary canals in three different quadrats of 1 mm^2^ of frontal skin samples from the affected animal and a control

		1st quadrat	2nd quadrat	3rd quadrat	Mean
Control	Hair count	9	10	9	9.3
	Mean diameter (μm)	89.9	85.1	86.6	87.2
	Total area (μm^2^)	59,689	59,274	56,286	58,416
Affected	Hair count	43	44	56	47.7
	Mean diameter (μm)	22.9	23.8	18.0	21.6
	Total area (μm^2^)	18,802	20,348	14,773	17,974

### Pedigree analysis

To better understand the genetic inheritance of this syndrome in the affected animal, we analyzed pedigree data and showed that with a coefficient of inbreeding of 0.0458, this calf was not more inbred than the Holstein calves born the same year (2014) with an average coefficient of inbreeding of 0.0459. We identified ten loops in the pedigree with common ancestors in the maternal and paternal lineages. Then, we predicted the number of inbred animals that were homozygous for alleles inherited from each of these common ancestors in the French Holstein population born from 2002 to 2017, which represents the putative number of affected animals born since the creation of the ONAB assuming a recessive inheritance. This number varied from 10 to 38,529 individuals depending on the ancestor (Additional file 1: Table S1). Considering that HED is a distinctive syndrome and that no other individual with a similar phenotype was reported in Holstein during this period, it is very unlikely that this case is due to a recessive mutation.

Thus, we investigated the hypothesis of a dominant inheritance associated with somatic or germline mosaicism, since both parents were clinically normal. The dam had only two other descendants, which were both unaffected. The sire had 9201 descendants and among these, none were reported to be affected by HED.

Finally, we estimated the mortality rate in the progeny of the sire of the affected calf. With 10.22% of calves that died between 0 and 6 months of age, its mortality rate is slightly lower than the average rate for the population (10.93%, +/− 0.21 sd). Thus, there is no evidence of increased mortality and of putative non reported HED calves among its progeny.

Based on these findings, we made the assumption that the mutation responsible for this isolated case of HED is a de novo and dominant mutation.

### Analysis of whole-genome sequences

In order to identify putative causal variants, we sequenced the whole genome of the affected calf. Based on the assumption of a de novo and dominant mutation, we retained only the heterozygous SNPs and small indels that were absent from the whole-genome sequencing data from 2331 bovine individuals in run 6 of the 1000 bull genomes project [18, 19]. We identified 5382 private heterozygous mutations among which only nine were predicted to affect the primary structure of a protein (Additional file 2: Table S2).

Six of these polymorphisms consisted of additions of one to three amino-acids in stretches of repeated amino-acids. The three remaining mutations were one putative deleterious missense mutation (SIFT score = 0) in the *solute carrier family 34 member 3 protein* (*SLC34A3* p.Y463D) gene and two frameshift mutations in the *suppressor of Ty 20 homolog* (*SUPT20H* p.Q642PfsX22) and the *phosphatidylinositol-4-phosphate 3-kinase C2 domain-containing beta* (*PIK3C2B* p.L441WfsX21) genes.

**Fig. 3:**
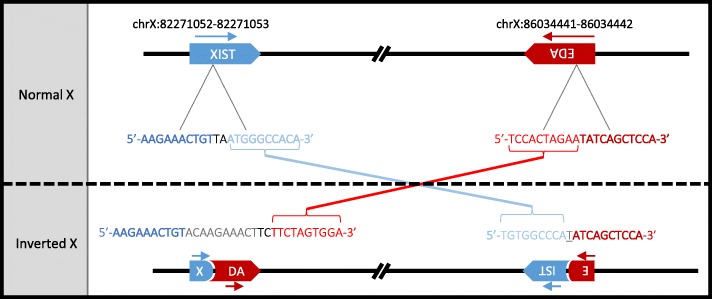
Scheme representing the de novo inversion between *XIST* and *EDA*. The first line provides information on the position of the breakpoints on the bovine UMD3.1 genome assembly. The nucleotides inserted in the first breakpoint after microhomology DNA repair are represented in grey while the nucleotide shared by both *XIST* and *EDA* segments at the second breakpoint is underlined

**Fig. 4:**
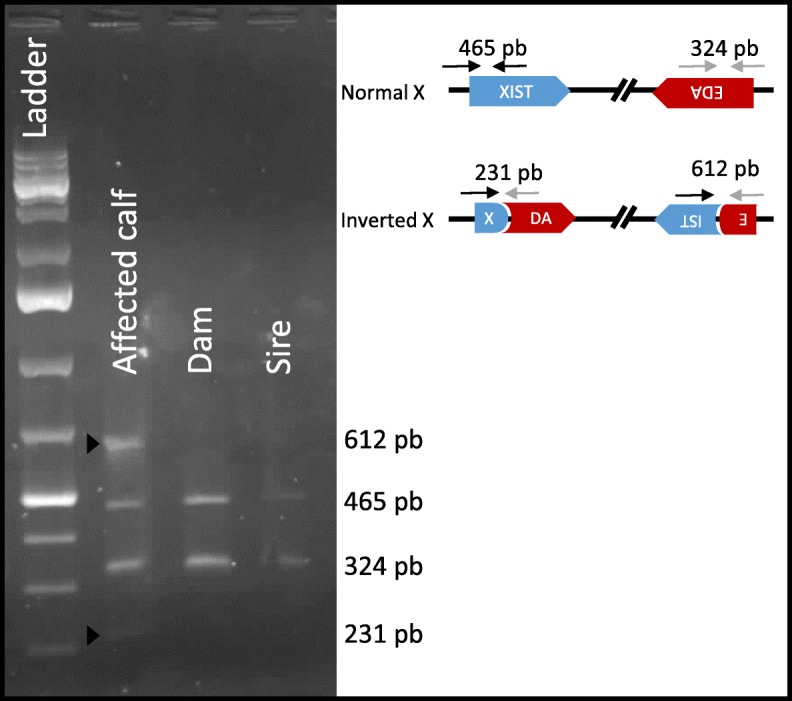
Genotyping of the de novo inversion using PCR and electrophoresis. Amplicons specific of the mutant allele (231 and 612 bp, in red) were observed only in the affected calf (see arrow-heads) while amplicons corresponding to the wild-type allele (324 and 465 bp) were amplified in the affected calf and both of its parents

Since with this approach, we investigated only the small variants that were located in coding regions [20], we then searched for structural variations in candidate genes using the Integrative Genomics Viewer (IGV) [21]. The study of *EDA*, *EDAR*, *EDARADD* and *WTN10* revealed a 3.8-Mb inversion with breakpoints located in the first intron of *EDA* and the first intron of *XIST* (Fig. 3, Additional file 8: Figure S4). Average sequencing depth in the region that encompasses the two breakpoints revealed no amplification or deletion of the inverted segment. (Additional file 9: Figure S5). PCR amplification of four segments that span these breakpoints in the mutant and wild-type alleles confirmed the existence and the de novo nature of this inversion. Indeed, only the affected calf was heterozygous for the mutation, whereas its parents were homozygous wild-type (Fig. 4). Subsequent Sanger sequencing of the PCR products enabled us to specify the nature of the nucleotide sequence at each breakpoint in the mutant allele. For the first breakpoint (between positions 82,271,052 and 82,271,053 bp on chromosome X, based on the bovine genome assembly UMD3.1), we observed a partial duplication of a 12-bp segment (5′-GTACAAGAAACT-3′), which suggested that microhomology-mediated break-induced replication [22] was involved in DNA repair. In contrast, for the second breakpoint, we observed a deletion of a single thymine (between positions 86,034,441 and 86,034,442 bp), which suggests that DNA repair resulted from non-homologous end-joining at this position. Finally, we analyzed 500-bp segments that encompass the breakpoints using dotplot [23] and identified no identical DNA sequence motif that could have promoted this inversion. Therefore, this mutation most probably results from a random break event.

### Immunostaining of EDA protein

To determine if a truncated EDA protein is expressed in the affected calf, we performed immunostaining in frontal skin tissue with antibodies specific for the EDA C-terminal and N-terminal ends. We observed relatively similar intensity and localization of staining in the case and control samples (Fig. 6; Additional file 11: Figure S7). However, the contrast of staining between the wall and the cytoplasm of the sebaceous glands cells was less marked in the affected animal than in the control with the antibody directed against the N-terminal region of the protein.

## Discussion

In this study, we explored an isolated case of a female Holstein calf with homogeneous hypotrichosis and oligodontia. Clinical and histopathological examinations revealed symptoms similar to those previously reported in *EDAR* homozygous and *EDA* hemizygous mutant cattle e.g. [5, 16, 17, 24], thus confirming the diagnosis of HED. In the affected animal, hair follicles were more numerous, thinner and located higher in the dermis of the frontal skin; sebaceous and sweat glands were hypoplastic; teeth were absent with the exception of one malformed premolar on each side of the upper jaw; while horn development appeared normal. This animal died at 2 weeks of age of pulmonary infection, probably aggravated by the absence of ciliae and mucous glands in the respiratory tract, as usually observed in HED [6, 14].

Analyzing the pedigree of the affected female, we identified ten ancestors present in both its paternal and maternal lineages. Nevertheless, this animal was not more inbred than the average of Holstein calves born the same year, with inbreeding coefficients of 4.58% versus 4.59%, respectively. By estimating the number of affected cases that would be expected if any of the common ancestors carried a recessive mutation, we concluded that, if they existed, other cases of HED should have been reported in France, which is not the case. Therefore, we assumed that the causal mutation is dominant and de novo.

Based on this hypothesis, we sequenced the whole genome of the affected calf, and conserved the small indels and heterozygous SNPs that were not observed in the population of 2331 bovine individuals of the 1000 bull genomes project [18, 19]. We identified nine variants that were predicted to affect the primary structure of a protein (Additional file 2: Table S2). Among them, six consist of additions of one to three amino acids in stretches of repeated amino acids. Since these stretches showed variation in size and/or poor conservation among orthologous proteins in mammals, we assumed that insertions at these sites are likely tolerated. The three remaining mutations are one putative deleterious missense mutation (SIFT score = 0) in the *SLC34A3* (p.Y463D) gene and two frameshift mutations in *SUPT20H* 5p.Q642PfsX22) and *PIK3C2B* (p.L441WfsX21). Mutations for knock-out alleles in these three genes have been well characterized in mouse. To our knowledge, no particular phenotype has been reported in heterozygous carrier animals, and none of the symptoms observed in homozygous individuals were consistent with HED. *SLC34A3* −/− mice have a modified calcium absorption and excretion [25, 26]. Some *SUPT20* −/− animals show major defects in the epithelial-to-mesenchymal transition during gastrulation, while others survive post-natally and appear normal [27]. Finally, *PIK3C2B* −/− mice were found to exhibit normal epidermal growth, differentiation and function in a study on the role of phosphoinositide 3-kinases in epidermal differentiation [28]. In conclusion, none of these nine SNPs and small indels appear to be relevant candidate mutations.

In addition, we conducted a search for structural variations in candidate genes (*EDA*, *EDAR*, *EDARADD* and *WTN10*) using IGV [21]. In doing so, we identified a large chromosomal inversion (3.8 Mb) with breakpoints located in the first intron of *EDA* and the first intron of *XIST* (Fig. 3, Additional file 8: Figure S4). Genotyping the case and its parents by PCR-electrophoresis allowed us to demonstrate the de novo origin of this mutation (Fig. 4).

*XIST* (X inactive specific transcript) encodes a long non-coding RNA that is responsible for the initiation of X-chromosome inactivation (XCI), i.e. the mechanism of dosage compensation that equalizes the expression of X-specific genes between genders in mammals. In female cells, one chromosome X is randomly inactivated, resulting in the expression of only one of the two sex chromosomes. The mechanism that regulates XCI is complex and not yet fully understood. XCI starts with the progressive coating of the future inactive chromosome by XIST RNA in cis, and involves a number of XIST activator [29] and repressor molecules [30] such as TSIX, a long non-coding RNA partially overlapping XIST and transcribed in the antisense orientation. In some cases, skewed-inactivation can occur due to negative or positive selection of one of the X chromosomes [7, 31, 32]. In humans and mouse, several mutations that affect the promoter or the coding regions of *XIST* are known to result in the preferential inactivation of the normal X chromosome in heterozygous females [33–35]. In parallel, analysis of a series of genome-edited human cultured cells demonstrated that partial deletions of *XIST* reactivate the inactive X chromosome [36]. While XCI is a conserved mechanism among mammals, the nature and importance of the *XIST* functional sites differ between man and mouse, the two species in which this mechanism has been most studied [36–38]. In agreement with this observation, analysis of Ensembl GERP conservation scores showed that, among the 91 eutherian mammals studied, the number of constrained elements in the *XIST* gene is small and these are mainly located in the region corresponding to human exon 4 (Additional file 10: Figure S6).

The inversion that we describe in this paper leads to the separation of the *XIST* exon 1 from the rest of the gene. Thus, if transcribed, the mutant XIST RNA would lack the main evolutionary constrained element among mammals, and contain only 48% of the normal transcript. In addition, this mutation does not affect the integrity of TSIX, which encodes the main repressor of XIST.

Although we could not conduct expression studies to validate our hypothesis, these arguments combined with the observation of a generalized HED phenotype in a female heterozygous for a mutation that truncates *EDA*, suggest that XCI is impaired on the X chromosome carrying the mutation and results in the skewed inactivation of the normal X.

In humans, several examples of females with X-linked HED due to complete skewed X-inactivation have been reported in the literature [7, 39, 40]. The patients were heterozygous carriers of a partial X-autosome translocation causing a disruption of the *EDA* gene. In these cases, the integrity of the *XIST* gene was not affected and the mechanism invoked to explain the skewed X inactivation was selective survival of embryonic cells inactivating the normal X. Indeed, inactivation of the derivative chromosome X is assumed to cause partial monosomy due to the extension of the inactivation wave onto the translocated autosome and is incompatible with further development [7]. In parallel, as previously mentioned, a number of mutations in the regulatory or coding regions of *XIST* have been reported to cause skewed inactivation and it is generally accepted that about 1% of females exhibit extremely *skewed* X-inactivation patterns i.e. 95:5 [41–43]. Nevertheless, cases of heterozygous females who carry mutations in *XIST* and in another chromosome X gene and are affected by a generalized syndrome, as observed here, are very uncommon [34, 44].

**Fig. 5:**
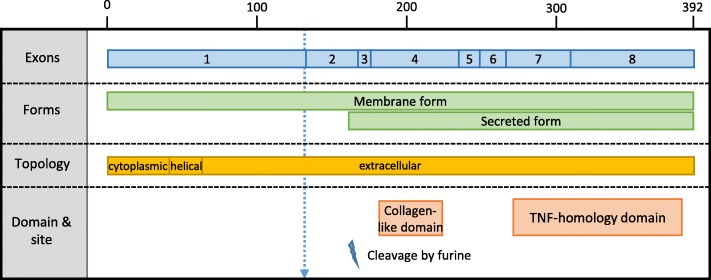
EDA isoforms and protein domains. Note that EDA-A1 and EDA-A2 are not separated since they only differ by two amino-acids in exon 8. Both of these isoforms possess a membrane form and a secreted form after cleavage by furine at position 159–160 (exon 2)

The second breakpoint is situated at position 86,034,441 bp within the *EDA* intron 1. *EDA* encodes two main transcripts EDA-A1 and EDA-A2 which differ only by two amino-acids (307 V and 308E localized in the TNF domain of the protein; exon 8). Both of these isoforms possess a membrane form and a secreted form after cleavage by furine at position 159–160 (exon 2). The extracellular part includes a collagen-like Gly-X-Y repeat that is essential for the trimerisation of the protein and a TNF-like signaling domain at the C-terminal end. The putative truncated protein would possess only the 132 first AA encoded by exon 1 and thus lack the cleavage site and the collagen-like and TNF-homology domains of the protein (Fig. 5). Many mutations in *EDA* have already been described in humans and mice, which provide insights on the impact of the deletion that we report in this article. Karlskov et al. [14] described a case of HED in cattle that was caused by a transcript variant containing a LINE1-derived pseudo exon between the first and the second exons of *EDA*. Since this pseudo exon results in a frameshift and a stop codon early in exon 2, we conclude that, in this HED case, the truncated protein encoded by only the first *EDA* exon would not be functional. In parallel, a complete deletion of the first exon has been reported to cause HED in humans [45] which supports the hypothesis that no putative alternative transcripts starting after exon 1 can compensate for the lack of the full transcripts. Thus, if expressed, the two parts of EDA that have been truncated by the inversion reported in this article are assumed to be non-functional.

**Fig. 6:**
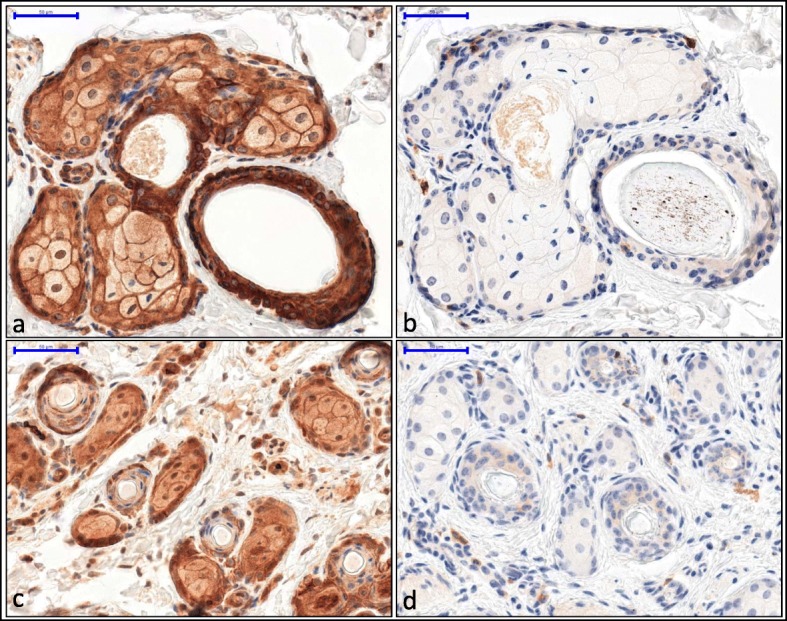
Immunostaining of the EDA protein in frontal tissue from control (**a, b**) and HED animals (**c, d**). Results of the N-ter immunostaining show a stronger contrast between cell wall and cytoplasm coloration in the control animal (**a**) than in the affected calf (**c**). Immunostaining of C-ter part of the protein appear similar between control (**b**) and affected (**d**) calves. Negative control of the C-ter is available in Additional file 11: Figure S7

Finally, to determine if a truncated EDA protein is expressed in the affected calf, we performed immunostaining with antibodies specific for the EDA C-terminal and N-terminal ends in frontal skin tissue. Immunostaining of the N-terminal end (Fig. 6a-c) revealed a relatively similar intensity and localization of staining in the case and control samples, thus supporting the existence of peptides containing the proximal part of the EDA protein in the skin of the affected calf. However, in a general manner, the contrast of staining between the wall and the cytoplasm of the sebaceous glands cells was less marked in the affected animal than in the control. While the region of EDA corresponding to exon 1 contains the helical domain required for membrane binding, it can be assumed that the lack of the C-terminal region affects its stability and/or transport to the cell wall. Surprisingly, immunostaining of the C-terminal part of the protein revealed the presence of peptides that correspond to the distal region of the EDA protein in the affected animals (Fig. 6b-d, Additional file 11: Figure S7). Considering the particular nature of the inversion and the transcription sense of the two broken genes, these peptides could be produced by a fusion transcript (e.g. [46]) that would start with the first exon of *XIST* and continue with exons 2 to 8 of *EDA*. Unfortunately, the lack of RNA material precluded further investigation. In any case, these analyses demonstrated that both parts of the EDA protein are expressed in spite of the rearrangement involving *EDA* and leading to a HED phenotype.

## Conclusions

In this study, we characterized a new mutation which is a strong candidate causal mutation for the hypohidrotic ectodermal dysplasia syndrome, that we detected in the female Holstein calf reported here. Mutations that occur in *EDA* on the X chromosome usually affect only the male carriers while the female carriers express few or no symptoms. The severe and homogeneous phenotype displayed by this animal could be explained by a preferential inactivation of the wild-type chromosome. Indeed, the affected calf carries an inversion that breaks both *EDA* and *XIST*, the keystone genes involved in X-inactivation. In spite of some limitation due to accessibility and conservation of biological samples, we present a body of evidence supporting the hypothesis that this mutation is responsible for a skewed inactivation of the X chromosomes, and that only the normal X can be inactivated. Based on our results, we present a new mode of inheritance for HED that may highlight the underlying mechanisms of dosage compensation when XIST is not functional.

## Methods

### Animals and sampling

The affected animal was photographed and reported by its breeder and veterinarian to the ONAB in its first week of life. It died the following week and we were informed of its death only 2 days later. In this context, we could obtain only a limited number of samples from the local veterinarian before disposal of the body. These specimens included formalin fixed samples from the frontal skin, the horn buds and the distal extremity of the lower jaw. Sampling for expression studies was not possible since RNA degrades in just a few hours after death. Blood samples for the female calf and its dam, as well as semen straws for the sire, were available for DNA extraction. Finally, we also sampled control tissues 6 h after death on a young Holstein calf that died of diarrhea.

### Histological analyses

Skin samples were fixed in 10% neutral buffered formalin for 24 h and embedded in paraffin wax according to standard histological protocols. Four-micrometer thick sections were mounted on adhesive slides (Klinipath- KP-PRINTER ADHESIVES). Paraffin-embedded sections were deparaffinized and stained with HES (haematoxylin, eosin and safran) to observe the skin morphology and with a Roan solution (kernechtrot, orange gelb and aniline blue) to study the hair follicles. Immunostaining for the C-terminal and N-terminal ends of EDA was performed using the Leica Bond RXm IHC staining system with the Bond Polymer Refined kit (DS9800), and antibodies against the EDA C-terminal (ab198022, abcam) and EDA N-terminal (OAAB03423, Aviva biosystem) ends, both diluted at 1:50. Incubation with the primary antibody and antigen retrieval were performed at pH = 9 for 20 min.

All slides were scanned with the Pannoramic Scan 150 (3D Histech) and analyzed with the CaseCenter 2.9 viewer (3D Histech). The number of hair follicles, their diameter and the total area covered by hair were measured in 1 mm^2^ quadrats on three different Roan-stained skin sections from the forehead region of the control and affected animals.

### Pedigree analysis

The average coefficient of inbreeding in the French population of Holstein females born in 2014 and in the affected animal were calculated with the program meow.f of the pedig package [47], which is based on the direct construction of the Cholesky decomposition of the kinship matrix. We used pedigree information from the French national databases.

The ancestors found in both the paternal and maternal lineages of the affected animal were identified based on pedigree data. Then, for each of these ancestors we calculated the expected numbers of affected animals in the French Holstein population assuming a recessive inheritance for HED. To do this, for any individual i in the population, we computed the contribution c (i) of this ancestor to i by gene dropping and then the probability of i of being homozygous for a mutation inherited from its ancestor as p_homo_(i) = c (sire) * c (dam) /4. Finally, we estimated the number of affected cases per year by summing the risk probabilities for all the calves born during the same period.

In addition, for 3577 bulls (including the sire of the HED female) with more than 100 descendants, we calculated the percentage of calves that died a natural death between zero and 6 months of age in their progeny.

### DNA extraction

Genomic DNA was extracted from blood and semen using the DNeasy Blood and Tissue Kit (Qiagen). DNA quality was controlled by electrophoresis and quantified using a Nanodrop spectrophotometer.

### Whole-genome sequencing

A paired-end library with an insert size of 450 bp was generated with the NEXTflex PCR-Free DNA Sequencing Kit (Bioscientific), and sequenced on a HiSeq 2000 sequencer (Illumina) with an average sequence coverage of 13.8x. Reads were mapped to the UMD3.1. bovine genome assembly using the Burrows Wheeler Aligner (BWA) tool [48]. Variants were called by using SAMtools 1.3.1 [49]. Assuming that the causative mutation is a dominant de novo mutation, all heterozygous variants detected for the affected calf were filtered against whole-genome sequence data from 2333 animals from more than 40 breeds that are part of the 1000 Bull Genomes Run 6.0 [19]. Private heterozygous SNPs and small indels with a quality score greater than 30 were annotated using the Ensembl Variant Effect Predictor pipeline (VeP) on the Ensembl v88 transcript set [50]. The SIFT software was used to predict the effect of non-synonymous coding variants [51]. Alignments of orthologous proteins among mammals were conducted using the CLUSTAL W (1.81) multiple sequence alignment tool implemented in Ensembl (www.ensembl.org) to evaluate the conservation of the amino-acids surrounding the sites of inframe insertions. Information on the domains that were missing in the proteins affected by frameshift mutations were obtained from the uniprot database (www.uniprot.org). Finally, the Integrative Genomics Viewer (IGV) [26] was used to search for structural variations within candidate genes (*EDA, EDAR, EDARADD and WTN10*) and 500-kb long regions up and downstream.

### Primer design and PCR

Four primers were designed to amplify segments that encompass the two breakpoints of the inversion based on the *Bos_taurus*_UMD_3.1/bosTau6 assembly (UCSC Genome Browser Gateway) using the Primer3 software [52]. Details on the PCR primers are in Additional file 3: Table S3. Four independent PCR reactions were performed to amplify the mutant and wild type alleles using the GO-Taq Flexi polymerase (Promega) according to the manufacturer’s instructions on a Mastercycle pro thermocycler (Eppendorf). Aliquots of PCR products were pooled for ethidium bromide-stained 2% agarose gel electrophoresis. The remaining amplicons were purified and sequenced by Eurofins (Eurofins MWG, Ebersberg Germany) using conventional Sanger sequencing.

### Alignment and annotation of *XIST*

The boundaries of the *XIST* exons were determined by alignment of the *XIST* sequence NR_001464.2 [38] with the BLAT tool of the UCSC genome browser on the UMD3.1 genome assembly [53], and comparative analysis of the human and cattle sequences of *XIST* made by Yen et al. [37]. The positions of the seven exons of the bovine XIST transcript are presented in Additional file 4: Table S4.

## Supplementary information

**Additional file 1: Table S1.** Number of expected calves with the same disease in the case of a recessive mutation.

**Additional file 2: Table S2.** Details on private heterozygous SNPs and small indels carried by the HED animal. QUAL and MQ scores: quality score of the variant and mapping quality score of the read containing the variant as calculated by SAMtools. DP4: number of reads containing the reference allele on the forward strand, the alternative allele on the forward strand, the reference allele on the reverse strand and the alternative allele on the reverse strand.

**Additional file 3: Table S3.** PCR primers for the amplification of segments spanning the inversion.

**Additional file 4: Table S4.** Position of *XIST* exons on the UMD3.1 bovine genome assembly.

**Additional file 5: Figure S1.** Phenotypic comparison of the affected calf (2 weeks, b-d-f) and a control (1 month, a-c-e). Clinical examination reveals abnormal eyelashes (a-b) but normal whiskers (c-d) and horn buds (e-f) in the affected versus control animal.

**Additional file 6: Figure S2.** Dental examination of the affected calf. X-ray of the proximal region of the lower jaw (a) reveals complete absence of deciduous and permanent incisors.

**Additional file 7: Figure S3.** Histological section of a horn bud from the affected animal. Note that the horn bud showed no notable morphological specificity. (PNG 472 kb)

**Additional file 8: Figure S4.** Visualization with IGV of the two breakpoints of the inversion affecting *XIST* (a) and *EDA* (b). Reads from pairs mapped with an aberrant orientation are color-coded to indicate the orientation of the second read of the pair (blue: reverse-reverse; green: forward-forward). Soft clipping parts of the reads are bright-colored.

**Additional file 9: Figure S5.** Plot of the average sequence depth in a 6-Mb interval encompassing the 3.8-Mb inverted segment. The sequence depth in the genomes of the affected animal (a) and a female control (b) was calculated for non-overlapping windows of 10 kb. Note the similarity of the profiles. A region that consisted of highly repeated centromeric satellites (ChrX:83151156–83,181,156) was removed for the sake of clarity (*). The localization of the inverted fragment is indicated with a double-headed arrow.

**Additional file 10: Figure S6.** Conservation of *XIST* exons (analyzed with Ensembl).

**Additional file 11: Figure S7.** Negative control of C-ter immunostaining in the affected calf. Negative control of C-ter immunostaining was performed on the affected animal without the primary antibodies. Note the complete absence of brown staining, which contrasts with the coloration in Fig. 6.

## Data Availability

The genome sequence of the affected calf was deposited in the Sequence Read Archive (SRA) (https://www.ncbi.nlm.nih.gov/sra) under the accession number: PRJNA534204. The genome sequence of the control female Holstein used for comparison of average sequencing depth is accessible under the accession number SAMEA3869561 in project PRJEB12703. The whole-genome sequence data of 2331 bovine individuals used for filtering is part of the 1000 Bull Genomes Run 6.0 [19]. More than half of these are already publicly available at accession numbers PRJNA238491, PRJEB9343, PRJNA176557, PRJEB18113, PRNJA343262, PRJNA324822, PRJNA324270, PRJNA277147, PRJEB5462. The complete dataset with all the variants of the 1000 Bull Genomes Run 6.0 used as control is available from the corresponding author on reasonable request.

## Abbreviations

BWA: Burrows Wheeler Aligner
*EDA*: *Ectodysplasin-A*
*EDAR*: *Ectodysplasin-A receptor*
*EDARRAD*: *EDAR associated death domain*
HED: Hypohidrotic Ectodermal Dysplasia
IGV: Integrative Genomics Viewer
INRA: French National Institute for Agricultural Research
ONAB: French National Observatory for Bovine Genetic Defects
PCR: Polymerase Chain Reaction
*PIK3C2B*: *Phosphatidylinositol-4-phosphate 3-kinase C2 domain-containing beta*
*SLC34A3*: *Solute carrier family 34 member 3 protein*
SNP: Single Nucleotide Polymorphism
SRA: Sequence Read Archive
*SUPT20H*: *suppressor of Ty 20 homolog*
TNF: Tumor necrosis Factor
VEP: Variant Effect Predictor
*WNT10A*: *Wingless-type mouse mammary tumor virus integration site family member 10A*
XCI: X-Chromosome Inactivation
*XIST*: *X inactive specific transcript*
XLHED: X-linked form of HED

## References

[1] Clarke A, Phillips DI (1987). Clinical aspects of X-linked hypohidrotic ectodermal dysplasia. Arch Dis Child.

[2] Lefebvre S, Mikkola M (2014). Ectodysplasin research—where to next?. Semin Immunol.

[3] Kuramoto T, Yokoe M, Hashimoto R, Hiai H, Serikawa T (2011). A rat model of hypohidrotic ectodermal dysplasia carries a missense mutation in the Edaradd gene. BMC Genet.

[4] Waluk D, Zur G, Kaufmann R, Welle M, Jagannathan V, Drögemüller C (2016). A splice defect in the EDA gene in dogs with an X-linked Hypohidrotic ectodermal dysplasia (XLHED) phenotype. G3: Genes|Genomes|Genetics.

[5] Drögemüller C, Kuiper H, Peters M, Guionaud S, Distl O, Leeb T (2002). Congenital hypotrichosis with anodontia in cattle: a genetic, clinical and histological analysis. Vet Dermatol.

[6] Seeliger F, Drögemüller C, Tegtmeier P, Baumgärtner W, Distl O, Leeb T (2005). Ectodysplasin-1 deficiency in a German Holstein bull associated with loss of respiratory mucous glands and chronic Rhinotracheitis. J Comp Pathol.

[7] Zankl A, Addor M-C, Cousin P, Gaide A-C, Gudinchet F, Schorderet D (2001). Fatal outcome in a female monozygotic twin with X-linked hypohydrotic ectodermal dysplasia (XLHED) due to a de novo t (X;9) translocation with probable disruption of the EDA gene. Eur J Pediatr.

[8] Cluzeau C, Hadj-Rabia S, Jambou M, Mansour S, Guigue P, Masmoudi S (2011). Only four genes (EDA1, EDAR, EDARADD, and WNT10A) account for 90% of hypohidrotic/anhidrotic ectodermal dysplasia cases. Hum Mutat.

[9] Tziotzios C, Petrof G, Liu L, Verma A, Wedgeworth EK, Mellerio JE (2014). Clinical features and WNT10A mutations in seven unrelated cases of Schöpf–Schulz–Passarge syndrome. Br J Dermatol.

[10] Gordon M, Nusse R (2006). Wnt signaling: multiple pathways, multiple receptors, and multiple transcription factors. J Biol Chem.

[11] Zeng B, Xiao X, Li S, Lu H, Lu J, Zhu L (2016). Eight mutations of three genes (EDA, EDAR, and WNT10A) identified in seven Hypohidrotic ectodermal dysplasia patients. Genes.

[12] Wiśniewski SAA, Kobielak A, Trzeciak WHH, Kobielak K (2002). Recent advances in understanding of the molecular basis of anhidrotic ectodermal dysplasia: discovery of a ligand, ectodysplasin a and its two receptors. J Appl Genet.

[13] Cui C, Schlessinger D (2006). EDA signaling and skin appendage development. Cell Cycle.

[14] Karlskov-Mortensen P, Cirera S, Nielsen OL, Arnbjerg J, Reibel J, Fredholm M (2011). Exonization of a LINE1 fragment implicated in X-linked hypohidrotic ectodermal dysplasia in cattle. Anim Genet.

[15] Cambiaghi S, Restano L, Pääkkönen K, Caputo R, Kere J (2000). Clinical findings in mosaic carriers of hypohidrotic ectodermal dysplasia. Arch Dermatol.

[16] Barlund CS, Clark EG, Leeb T, Drögemüller C, Palmer CW (2007). Congenital hypotrichosis and partial anodontia in a crossbred beef calf. Can Vet J.

[17] Ogino A, Kohama N, Ishikawa S, Tomita K, Nonaka S, Shimizu K (2011). A novel mutation of the bovine EDA gene associated with anhidrotic ectodermal dysplasia in Holstein cattle. Hereditas.

[18] Daetwyler H, Capitan A, Pausch H, Stothard P, van BR, Brøndum R (2014). Whole-genome sequencing of 234 bulls facilitates mapping of monogenic and complex traits in cattle. Nat Genet.

[19] Bouwman A, Daetwyler H, Chamberlain A, Ponce C, Sargolzaei M, Schenkel F (2018). Meta-analysis of genome-wide association studies for cattle stature identifies common genes that regulate body size in mammals. Nat Genet.

[20] Michot P, Fantini O, Braque R, Allais-Bonnet A, Saintilan R, Grohs C (2015). Whole-genome sequencing identifies a homozygous deletion encompassing exons 17 to 23 of the integrin beta 4 gene in a Charolais calf with junctional epidermolysis bullosa. Genet Sel Evol.

[21] Robinson J, Thorvaldsdóttir H, Winckler W, Guttman M, Lander E, Getz G (2011). Integrative genomics viewer. Nat Biotechnol.

[22] Hastings IG, Lupski J (2009). A microhomology-mediated break-induced replication model for the origin of human copy number variation. PLoS Genet.

[23] Sonnhammer E, Durbin R (1995). A dot-matrix program with dynamic threshold control suited for genomic DNA and protein sequence analysis. Gene.

[24] Bourneuf E, Otz P, Pausch H, Jagannathan V, Michot P, Grohs C (2017). Rapid discovery of De novo deleterious mutations in cattle enhances the value of livestock as model species. Sci Rep.

[25] Segawa H, Onitsuka A, Kuwahata M, Hanabusa E, Furutani J, Kaneko I (2009). Type IIc sodium–dependent phosphate transporter regulates calcium metabolism. J Am Soc Nephrol.

[26] Myakala K, Motta S, Murer H, Wagner C, Koesters R, Biber J (2014). Renal-specific and inducible depletion of NaPi-IIc/Slc34a3, the cotransporter mutated in HHRH, does not affect phosphate or calcium homeostasis in mice. Am J Physiol-renal.

[27] Zohn I, Li Y, Skolnik E, Anderson K, Han J, Niswander L (2006). p38 and a p38-interacting protein are critical for downregulation of E-cadherin during mouse gastrulation. Cell.

[28] Harada K, Truong AB, Cai T, Khavari PA (2005). The class II phosphoinositide 3-kinase C2β is not essential for epidermal differentiation. Mol Cell Biol.

[29] Barakat T, Jonkers I, Monkhorst K, Gribnau J (2010). X-changing information on X inactivation. Exp Cell Res.

[30] Senner C, Brockdorff N (2009). Xist gene regulation at the onset of X inactivation. Curr Opin Genet Dev.

[31] Renault N, Dyack S, Dobson M, Costa T, Lam W, Greer W (2007). Heritable skewed X-chromosome inactivation leads to haemophilia a expression in heterozygous females. Eur J Hum Genet.

[32] Belmont JW (1996). Genetic control of X inactivation and processes leading to X-inactivation skewing. Am J Hum Genet.

[33] Clerc P, Avner P (1998). Role of the region 3′ to Xist exon 6 in the counting process of X-chromosome inactivation. Nat Genet.

[34] Plenge RM, Hendrich BD, Schwartz C, Arena JF, Naumova A, Sapienza C (1997). A promoter mutation in the XIST gene in two unrelated families with skewed X-chromosome inactivation. Nat Genet.

[35] Marahrens Y, Panning B, Dausman J, Strauss W, Jaenisch R (1997). Xist-deficient mice are defective in dosage compensation but not spermatogenesis. Genes Dev.

[36] Lee HJ, Gopalappa R, Sunwoo H, Choi S-WW, Ramakrishna S, Lee JT (2019). En bloc and segmental deletions of human XIST reveal X chromosome inactivation-involving RNA elements. Nucleic Acids Res.

[37] Yen Z, Meyer I, Karalic S, Brown C (2007). A cross-species comparison of X-chromosome inactivation in Eutheria. Genomics.

[38] Chureau C, Prissette M, Bourdet A, Barbe V, Cattolico L, Jones L (2002). Comparative sequence analysis of the X-inactivation center region in mouse, human, and bovine. Genome Res.

[39] Kere J, Grzeschik KH, Limon J, Genomics G-M (1993). Anhidrotic ectodermal dysplasia gene region cloned in yeast artificial chromosomes. Genomics.

[40] MacDermot KD, genetics H-M (1990). Female with hypohidrotic ectodermal dysplasia and de novo (X; 9) translocation. Hum Genet.

[41] Gale RE, Wheadon H, Boulos P, Blood L-D (1994). Tissue specificity of X-chromosome inactivation patterns. Blood.

[42] Naumova AK, Plenge RM, Bird LM, Leppert M, Morga K, Willard HF, Sapienza C (1996). Heritability of X chromosome--inactivation phenotype in a large family. Am J Hum Genet.

[43] Allen RC, Zoghbi HY, Moseley AB, Rosenblatt HM, Belmont JW (1992). Methylation of HpaII and HhaI sites near the polymorphic CAG repeat in the human androgen-receptor gene correlates with X chromosome inactivation. Am J Hum Genet.

[44] Pereira L, Zatz M (1999). Screening of the C43G mutation in the promoter region of the XIST gene in females with highly skewed X-chromosome inactivation. Am J Med Genet.

[45] Pääkkönen K, Cambiaghi S, Novelli G, Ouzts L, Penttinen M, Kere J (2001). The mutation spectrum of the EDA gene in X-linked anhidrotic ectodermal dysplasia. Hum Mutat.

[46] Puig M, Castellano D, Pantano L, Giner-Delgado C, Izquierdo D, Gayà-Vidal M (2015). Functional impact and evolution of a novel human polymorphic inversion that disrupts a gene and Creates a fusion transcript. PLoS Genet.

[47] Boichard D (2002). PEDIG: a fortran package for pedigree analysis suited for large populations. 7th world congress on genetics applied to livestock production.

[48] Li H, Durbin R (2009). Fast and accurate short read alignment with burrows–wheeler transform. Bioinformatics.

[49] Li H, Handsaker B, Wysoker A, Fennell T, Ruan J, Homer N (2009). The sequence alignment/map format and SAMtools. Bioinformatics.

[50] McLaren W, Pritchard B, Rios D, Chen Y, Flicek P, Cunningham F (2010). Deriving the consequences of genomic variants with the Ensembl API and SNP effect predictor. Bioinformatics.

[51] Kumar P, Henikoff S, Ng P (2009). Predicting the effects of coding non-synonymous variants on protein function using the SIFT algorithm. Nat Protoc.

[52] Untergasser A, Cutcutache I, Koressaar T, Ye J, Faircloth B, Remm M (2012). Primer3—new capabilities and interfaces. Nucleic Acids Res.

[53] Kent W (2002). BLAT—the BLAST-like alignment tool. Genome Res.

