# Supplementary material for: Α de novo 3.8-Mb inversion affecting the EDA and XIST genes in a heterozygous female calf with generalized hypohidrotic ectodermal dysplasia
Source: BMC Genomics. 2019 Sep 18;20:715. doi: 10.1186/s12864-019-6087-1 (PMC6749632; doi:10.1186/s12864-019-6087-1)
Supplement: Supplementary file 1 — Additional file 1: Table S1. Number of expected calves with the same disease in the case of a recessive mutation. [file 12864_2019_6087_MOESM1_ESM.docx]

| International ID | Name | Birth  year | Nb of inbred descendants | Exp. nb of cases |
| --- | --- | --- | --- | --- |
| HOLCANM000000267150 | CITATION R | 1958 | 6543451 | 41 |
| HOLCANM000000275932 | ROCKMAN S | 1960 | 7861676 | 148 |
| HOLCANM000000299855 | ROYAL MARK | 1966 | 916479 | 34 |
| HOLCANM000000383622 | AEROSTAR | 1985 | 5861293 | 11911 |
| HOLUSAM000001483844 | HAPPY | 1965 | 1449015 | 10 |
| HOLUSAM000001629391 | TRIPLETHREAT | 1972 | 4161669 | 341 |
| HOLUSAM000001650414 | VALIANT | 1973 | 19506718 | 38529 |
| HOLUSAM000001773417 | MARK | 1978 | 8556678 | 7497 |
| HOLUSAM000001912270 | ELTON | 1983 | 5125175 | 6929 |
| HOLUSAM000001929410 | BLACKSTAR | 1983 | 8832675 | 13782 |
