# Supplementary material for: Α de novo 3.8-Mb inversion affecting the EDA and XIST genes in a heterozygous female calf with generalized hypohidrotic ectodermal dysplasia
Source: BMC Genomics. 2019 Sep 18;20:715. doi: 10.1186/s12864-019-6087-1 (PMC6749632; doi:10.1186/s12864-019-6087-1)
Supplement: Supplementary file 3 — Additional file 3: Table S3. PCR primers for the amplification of segments spanning the inversion. [file 12864_2019_6087_MOESM3_ESM.docx]

| **egions** | **Alleles** | **Primer pairs** | **Size of amplicons**  **(bp)** |
| --- | --- | --- | --- |
| First breakpoint | Wt | CCGATCCAGGAATCGAACTA/  AAGGCACTGAGGGTTTAGCA | 324 |
| Second breakpoint | Wt | CATTACCCCAGAGTGCACAA/  TGAGGTGGGAACAGGATTTT | 465 |
| First breakpoint | Mutant | CCGATCCAGGAATCGAACTA / ATGCACTGCTGTCCTTTTTG | 612 |
| Second breakpoint | Mutant | TGAGGTGGGAACAGGATTTT / AAGGCACTGAGGGTTTAGCA | 231 |
