# Supplementary material for: Α de novo 3.8-Mb inversion affecting the EDA and XIST genes in a heterozygous female calf with generalized hypohidrotic ectodermal dysplasia
Source: BMC Genomics. 2019 Sep 18;20:715. doi: 10.1186/s12864-019-6087-1 (PMC6749632; doi:10.1186/s12864-019-6087-1)
Supplement: Supplementary file 4 — Additional file 4: Table S4. Position of XIST exons on the UMD3.1 bovine genome assembly. [file 12864_2019_6087_MOESM4_ESM.docx]

| Exon number | Position |
| --- | --- |
| Exon 1 | ChrX:82261155-82270520 |
| Exon 2 | ChrX:82275727-82278233 |
| Exon 3 | ChrX:82279636-82279727 |
| Exon 4 | ChrX:82281202-82281339 |
| Exon 5 | ChrX:82282865-82282008 |
| Exon 6 | ChrX:82286074-82286205 |
| Exon 7 | ChrX:82287249-82294467 |
