# Supplementary figures and images for: Α de novo 3.8-Mb inversion affecting the EDA and XIST genes in a heterozygous female calf with generalized hypohidrotic ectodermal dysplasia
Source: BMC Genomics. 2019 Sep 18;20:715. doi: 10.1186/s12864-019-6087-1 (PMC6749632; doi:10.1186/s12864-019-6087-1)

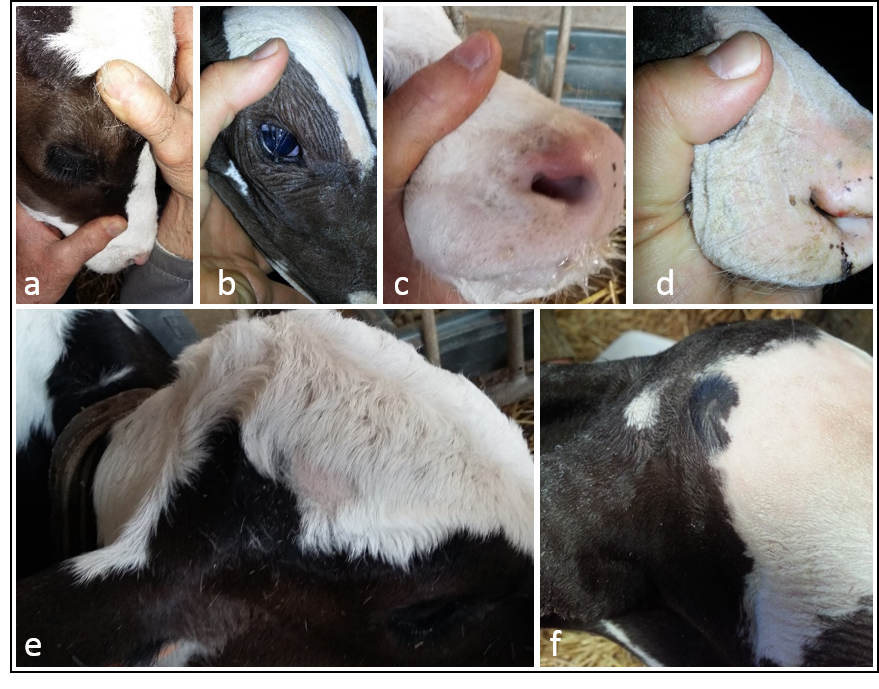

Supplement: Supplementary file 5 — Additional file 5: Figure S1. Phenotypic comparison of the affected calf (2 weeks, b-d-f) and a control (1 month, a-c-e). Clinical examination reveals abnormal eyelashes (a-b) but normal whiskers (c-d) and horn buds (e-f) in the affected versus control animal. [file 12864_2019_6087_MOESM5_ESM.png]

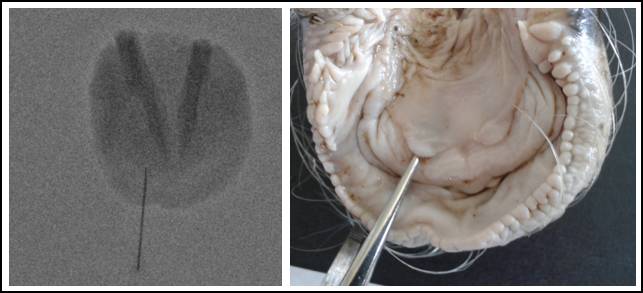

Supplement: Supplementary file 6 — Additional file 6: Figure S2. Dental examination of the affected calf. X-ray of the proximal region of the lower jaw (a) reveals complete absence of deciduous and permanent incisors. [file 12864_2019_6087_MOESM6_ESM.png]

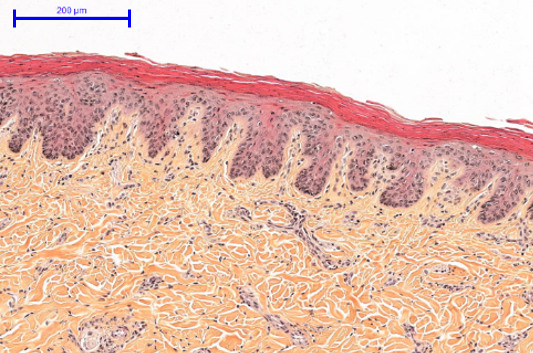

Supplement: Supplementary file 7 — Additional file 7: Figure S3. Histological section of a horn bud from the affected animal. Note that the horn bud showed no notable morphological specificity. (PNG 472 kb) [file 12864_2019_6087_MOESM7_ESM.png]

## Slide 1
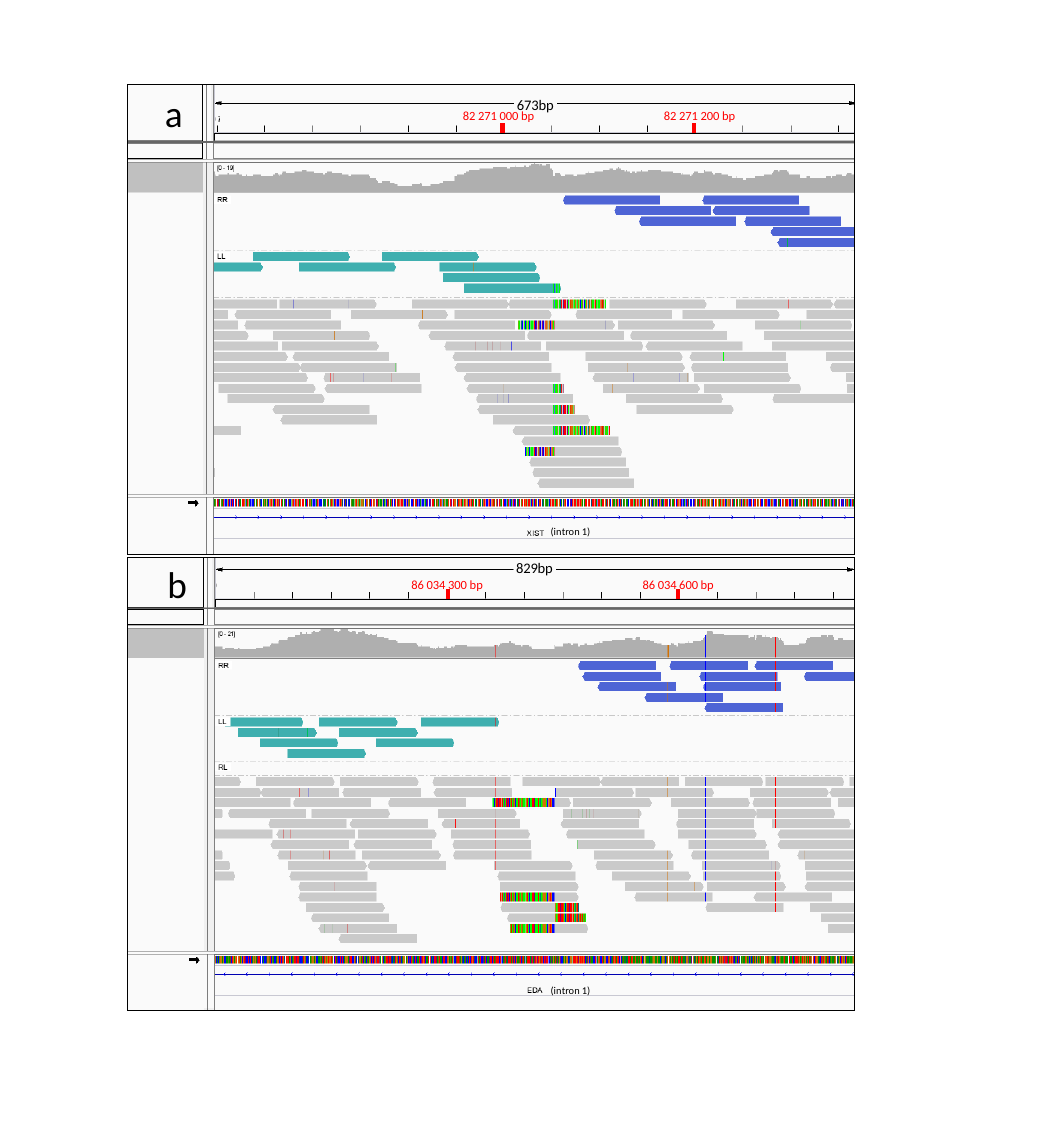

a
673bp
82 271 000 bp
82 271 200 bp
(intron 1)
829bp
86 034 300 bp
86 034 600 bp
(intron 1)
b

Supplement: Supplementary file 8 — Additional file 8: Figure S4. Visualization with IGV of the two breakpoints of the inversion affecting XIST (a) and EDA (b). Reads from pairs mapped with an aberrant orientation are color-coded to indicate the orientation of the second read of the pair (blue: reverse-reverse; green: forward-forward). Soft clipping parts of the reads are bright-colored. [file 12864_2019_6087_MOESM8_ESM.pptx]

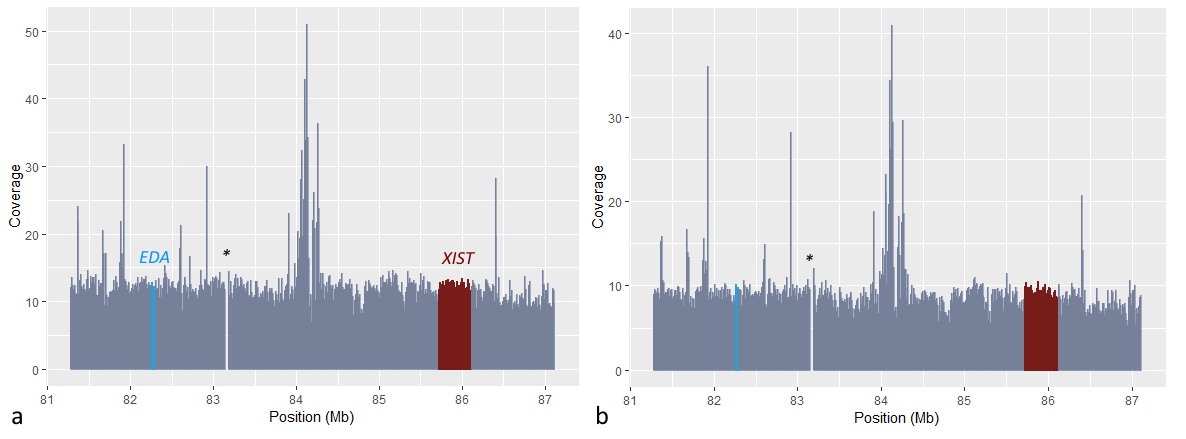

Supplement: Supplementary file 9 — Additional file 9: Figure S5. Plot of the average sequence depth in a 6-Mb interval encompassing the 3.8-Mb inverted segment. The sequence depth in the genomes of the affected animal (a) and a female control (b) was calculated for non-overlapping windows of 10 kb. Note the similarity of the profiles. A region that consisted of highly repeated centromeric satellites (ChrX:83151156–83,181,156) was removed for the sake of clarity (*). The localization of the inverted fragment is indicated with a double-headed arrow. [file 12864_2019_6087_MOESM9_ESM.png]

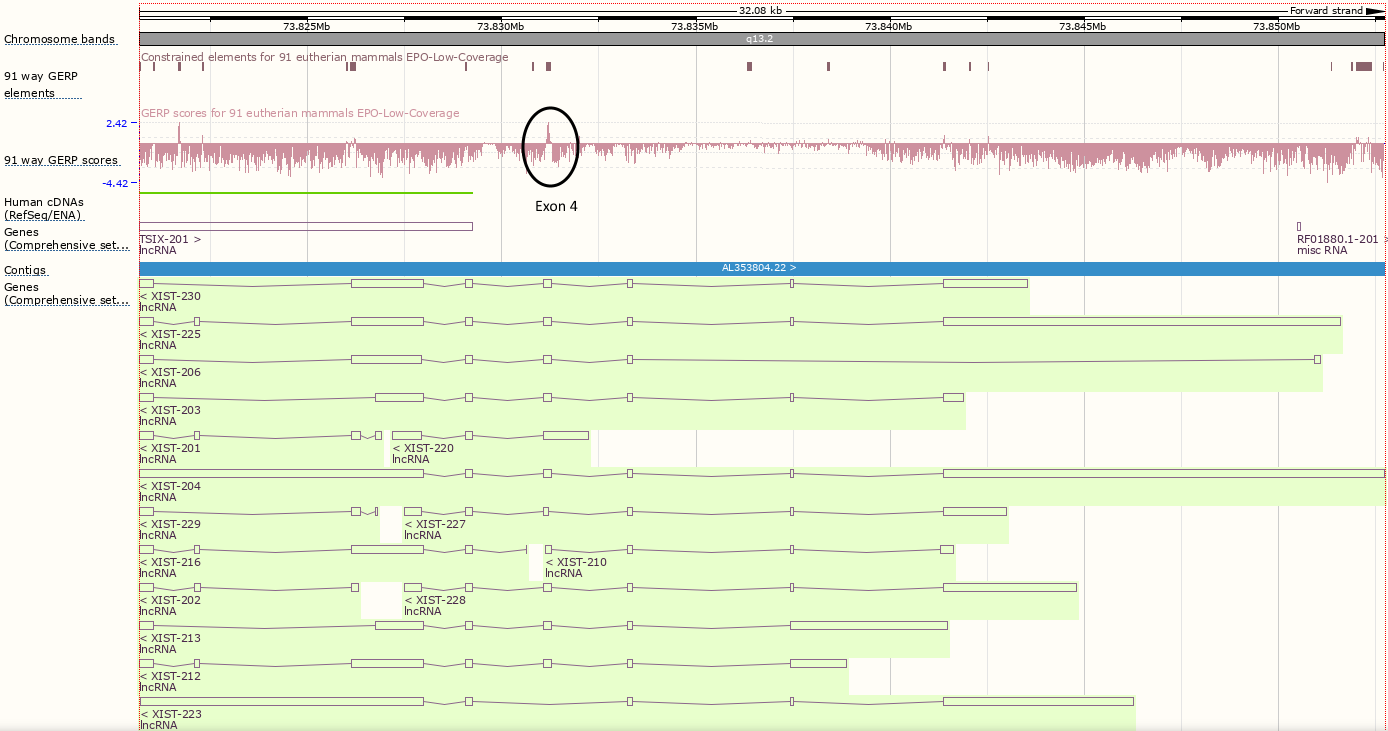

Supplement: Supplementary file 10 — Additional file 10: Figure S6. Conservation of XIST exons (analyzed with Ensembl). [file 12864_2019_6087_MOESM10_ESM.png]

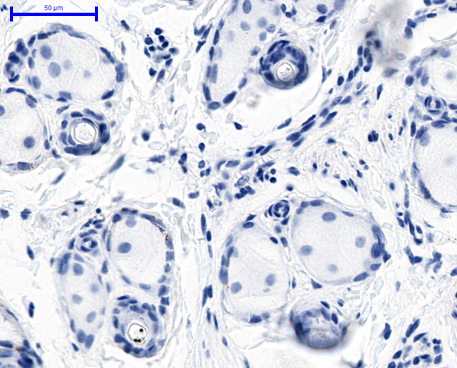

Supplement: Supplementary file 11 — Additional file 11: Figure S7. Negative control of C-ter immunostaining in the affected calf. Negative control of C-ter immunostaining was performed on the affected animal without the primary antibodies. Note the complete absence of brown staining, which contrasts with the coloration in Fig. 6. [file 12864_2019_6087_MOESM11_ESM.png]
